# Clinician perceptions and patient experiences of antiretroviral treatment integration in primary health care clinics, Tshwane, South Africa

**DOI:** 10.4102/curationis.v38i1.1489

**Published:** 2015-10-02

**Authors:** Maphuthego D. Mathibe, Stephen J.H. Hendricks, Anne-Marie Bergh

**Affiliations:** 1 School of Health Systems and Public Health, University of Pretoria, South Africa; 2 Health and Social Development & SRAC, City of Tshwane, South Africa; 3 MRC Unit for Maternal and Infant Health Care Strategies, University of Pretoria, South Africa

## Abstract

**Background:**

Primary Health Care (PHC) clinicians and patients are major role players in the South African antiretroviral treatment programme. Understanding their perceptions and experiences of integrated care and the management of people living with HIV and AIDS in PHC facilities is necessary for successful implementation and sustainability of integration.

**Objective:**

This study explored clinician perceptions and patient experiences of integration of antiretroviral treatment in PHC clinics.

**Method:**

An exploratory, qualitative study was conducted in four city of Tshwane PHC facilities. Two urban and two rural facilities following different models of integration were included. A self-administered questionnaire with open-ended items was completed by 35 clinicians and four focus group interviews were conducted with HIV-positive patients. The data were coded and categories were grouped into sub-themes and themes.

**Results:**

Workload, staff development and support for integration affected clinicians’ performance and viewpoints. They perceived promotion of privacy, reduced discrimination and increased access to comprehensive care as benefits of service integration. Delays, poor patient care and patient dissatisfaction were viewed as negative aspects of integration. In three facilities patients were satisfied with integration or semi-integration and felt common queues prevented stigma and discrimination, whilst the reverse was true in the facility with separate services. Single-month issuance of antiretroviral drugs and clinic schedule organisation was viewed negatively, as well as poor staff attitudes, poor communication and long waiting times.

**Conclusion:**

Although a fully integrated service model is preferable, aspects that need further attention are management support from health authorities for health facilities, improved working conditions and appropriate staff development opportunities.

## Introduction

Worldwide 35.3 million people over the age of 15 years were infected with HIV in 2012, whilst sub-Saharan Africa accounted for 70% of all new HIV infections in the same year. South Africa had an adult HIV prevalence of 17.9%, with about 6.1 million people living with the virus in 2012 (United Nations Programme on HIV/AIDS [UNAIDS]^2013^:4). According to the 2011 Antenatal HIV Prevalence Survey, Gauteng province had an HIV prevalence rate of 28.7% and Tshwane district a rate of 24.4% (National Department of Health [NDOH] 2012a:14, 17).

Accessing treatment remains a challenge for people living with HIV and AIDS (PLWHA). Only 49% of eligible people had access to antiretroviral treatment (ART) in sub-Saharan Africa in 2010 (United States Agency for International Development [USAID] 2012:4). In South Africa, the majority of people in need of treatment were located in the public sector in 2010 (Smith *et al.* 2011; UNAIDS 2013). ART services in South Africa started in 2004 at accredited hospitals and non-governmental organisations (NGOs). The services were vertical and doctor driven and had separate dedicated resources (Uebel et al. 2013a:2).

Owing to the increasing demand for treatment, the NDOH decentralised ART services to primary health care (PHC) facilities. This decision was announced on 01 December 2009 by President Zuma (Presidency 2009). By May 2011, 2205 PHC facilities provided ART services and by March 2013 the number had increased to over 3500 clinics, as compared to the 490 facilities of 2010 (Conradie 2014:6; Motsoaledi 2011).

### Background

Integration of ART services into PHC remains a priority of government in order to provide efficient services, strengthen resources and improve access to ART for PLWHA (Uebel et al. 2013a:2). Uebel et al. (2013a:9) reported that models of integration for HIV care ‘varied on a spectrum from a fully integrated service, where patients accessed HIV care from any nurse, to a more separate delivery of care, with patients accessing care from a specific ART nurse’. Sibiya and Gwele (2009:35) describe integrated comprehensive PHC services as a supermarket or one-stop approach. For the purpose of this report the NDOH's (2012b:9) definition of ‘integration’ will be used, referring to a ‘one-stop shop under one roof’ service to ensure comprehensive health care services to patients.

The main challenges faced by the NDOH are ensuring the availability of resources in terms of infrastructure, human resources and an adequate skills mix, and management capabilities for coping with the provision of comprehensive PHC services and care for the increasing number of PLWHA (Askew & Berer 2003:55; Davies, Homfray & Venables 2013). The PHC practitioners have to maintain high quality care services whilst trying to cope with increased uptake of treatment, as more people become in need of lifelong ART (Crowley & Stellenberg 2014:616; Van der Merwe et al. 2006:579; Van Rensburg et al. 2008:2; Wanderler et al. 2012:14).

Poor service delivery, heavy workload, high staff turnover, time constraints, resource gaps and uneven distribution of resources have been reported as common problems in ART services in PHC clinics (Avert 2009; Chehab et al. 2011:4; Cullinan 2013:1; Davies et al. 2013:3–6; UNAIDS 2013). Askew and Berer (2003:55) pointed out that expecting health workers to bear additional activities relating to HIV might be overburdening, frustrating to nurses and not feasible. These challenges require sufficient strengthening of support and training (Crowley & Stellenberg 2014:616; Uebel et al. 2013a:2).

The Health Systems Trust (HST 2010) emphasised that strengthening management systems at PHC level would improve effective utilisation of available resources. Active involvement of support staff to cope with human-resource demands for the provision of HIV and AIDS care at PHC level and scaling up ART services was necessary (Harrison 2009:28). Some countries have also implemented the task-shifting guidelines of the World Health Organisation (WHO 2007) to address health worker shortages (Callaghan, Ford & Schneider 2010; Ruud et al. 2010:417).

A few studies regarding service delivery for PLWHA have been conducted in the Tshwane Metro area of Gauteng province, South Africa. In a study assessing the quality of ART services in public PHC clinics, the clinicians’ consultation quality scored the lowest in all the health facilities included in the study and it was found that clinicians did physical examinations in only 41% of client visits (Kinkel et al. 2012:1). Louwagie et al. (2012:1052) compared access to HIV-related care between non-integrated facilities (ART in separate facilities) and semi-integrated facilities (one roof, different rooms and different clinicians) and found that a higher percentage of patients were initiated on ART in the latter facilities than in the former.

Managers’ and nurses’ preference on how to render services and patients’ preference of care are the main factors found to have affected the effort to integrate ART into PHC services (Uebel et al. 2013a). From the researchers’ observations in Tshwane, PHC facilities responded to the challenge of ART-service provision in three different ways. Some facilities were proactive and reorganised the services in their facilities to fully integrate ART services into the PHC package. A second group of facilities accommodated the service in a separate room or structure. The third group of facilities demonstrated resistance to embrace integration, citing various reasons ranging from space to human resources, despite directives for the roll-out of ART services. Elsewhere it has been found that the attitudes of facility managers towards the ART programme roll-out in PHC facilities play an important role in ensuring successful implementation (Davies et al. 2013).

### Problem statement and research objectives

Although some previous studies assessed the quality of ART services in public hospitals and clinics (Kinkel et al. 2012; Louwagie et al. 2012; Mabitsi 2013), extensive reports on the experiences and perceptions of clinicians regarding ART integration in PHC clinics were not available at the time of this study. More information in this area was important for establishing the nature of challenges associated with the provision of long-term integrated outpatient care to many PLWHA requiring lifelong management and monitoring. The success and sustainability of the massive South African ART programme relies on understanding the perspectives of clinical staff and the experiences of patients regarding ART programme implementation, strengthening management decisions relating to resource mobilisation, and quality improvement efforts in PHC facilities. Based on the imperative of scaling up and integrating ART services into the PHC package a need was identified for answering the question, ‘What are PHC clinicians’ perceptions and patients’ experiences of the integration of ART services into the comprehensive PHC package in Tshwane health facilities?’ more systematically.

#### Objective of the study

The objective of the study was to describe clinicians’ perceptions and patients’ experiences of integrated ART services in PHC clinics and to recommend strategies for the improvement of PHC services.

## Methods

### Design

An exploratory qualitative descriptive study design was used to gain insight into clinicians’ perspectives and to learn about patients’ experiences of ART integration into comprehensive PHC.

### Context of the study and study population

The study was conducted in four PHC health facilities in the Tshwane district of Gauteng. Three were clinics providing services five or six days per week during office hours and one was a community health centre providing a 24-hour service.

Sampling was carried out in two phases. Firstly, the research sites were identified and, after that, research participants were selected from those sites.

**FIGURE 1:**
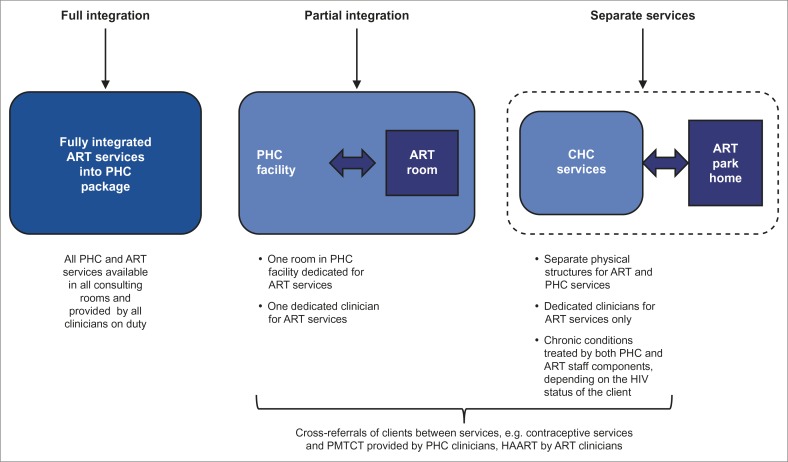
Antiretroviral treatment service integration models at primary health care PHC facilities.

As this was an exploratory study, the study facilities were purposively selected. It was anticipated that studying four facilities with attendance of more than 6000 patients per month and implementing different systems of integration of services would provide sufficient insights to fulfil the study objectives. A distinction was made between fully integrated, partially integrated and separate services (Figure 1). Two facilities were fully integrated and one partially integrated; one facility had separate services. The sample included two urban and two rural facilities to ensure geographical spread. One rural facility with a patient headcount of around 2500 per month did not meet the criterion of > 6000 patients per month, but was included because of its system of fully integrated services. Two facilities were managed by Tshwane Metro Health Department and two by the Gauteng Provincial Health Department.

In the second phase, clinicians and patients from the selected facilities were sampled. The clinician population consisted of the total number of clinicians (enrolled nurses, professional nurses and doctors) working in the four facilities to complete a questionnaire. In each of the four facilities a convenient sample of consenting HIV-positive patients older than 18 years was recruited consecutively for participation in a focus group interview.

### Data collection

Data collection took place in the first half of 2013.

#### Clinician questionnaire

A self-administered, anonymous questionnaire with open-ended items was distributed to almost all clinicians in the four facilities. Thirty-five of 42 clinicians returned the completed questionnaire; most participants were professional nurses, with one doctor and one enrolled nurse. Clinicians’ perspectives were elicited in the questionnaire on the following: organisation and integration of ART services in the health facility; issues affecting the quality of ART services; support for ART services from management, colleagues and others; positive aspects of and challenges associated with ART integration; and suggestions for improvement of ART service integration in the facility. Because the questionnaire was completed anonymously, individual views could not be probed further. However, a fairly large number of clinicians participated in completing the questionnaire and the views expressed by different healthcare providers complemented each other or confirmed a particular perception.

#### Focus group interviews with patients

Focus group (FG) interviews were conducted with willing clients attending the ART service in the four facilities. One FG was held in each facility. The interviews explored patient experiences with integrated ART services and perceptions of the quality of services in the particular health facility. A semi-structured interview guide was used and topics included the following: care and support received from health workers; issues affecting ART service quality; accessibility of services to PLWHA in the community; and suggestions for improving services. All interviews were audio-recorded.

The FG interviews ranged between 20 minutes and one hour 15 minutes and continued until no new views were expressed. The duration also depended on the number of participants in each group. Participants ranged from two to eight participants per group and all groups included males and females. The interviews in the two urban facilities were conducted in English because of the variety of first languages spoken. Explanations were also given in any of the local languages where needed. The interviews in the rural facilities were conducted in Sepedi by the first author, who is a first language Sepedi speaker with experience of the different ART service integration models.

### Data analysis

Each completed questionnaire and each FG transcript received a unique code for filing purposes. The open-ended answers from the clinician questionnaires were typed in a table format to facilitate comparison between different participant views. All patient FG interviews were transcribed in the language in which they had been conducted (English or Sepedi) and the first analysis was also conducted in the same language. Selected quotations from the Sepedi FG transcripts were later translated to English for further use.

The data from the clinicians’ open-ended responses and the transcripts of the client FGs were initially read repeatedly in order for the researchers to become immersed in the data. Subsequently the data were coded and categories grouped into sub-themes and themes related to clinician perceptions and client experiences (Liamputtong & Ezzy 2005).

Direct quotations in this report are provided with codes to identify the facility to which participants were linked. Clinician participant quotations are only identified by the codes F1, F2, F3 or F4, according to facility. Patient participants are identified by gender and facility. Female participants are referred to by codes FF1, FF2, FF3 or FF4. Male participants have the codes MF1, MF2, MF3 or MF4. Facilities 1 and 2 (F1 and F2) provided fully integrated services. Facility 3 (F3) provided partially integrated services and facility 4 (F4) separate services (Figure 1).

## Results

The findings are divided under two main headings, clinician perceptions, and patient experiences. The main themes, sub-themes and categories emerging from the analysis are presented for clinician and patient participants in Tables 1 and 2 respectively.

**TABLE 1:** Clinician perceptions of factors affecting integration and quality of care.

Themes	Sub-themes	Categories
Provider-related factors and work environment	Workload	Adequacy of staff Increased activities Across-border clients Down-referrals
	Staff development	Skills ART guideline knowledge
	Support for integration	Space/ infrastructure Resources Support staff Staff interest and mutual support
Integration factors affecting client services	Favourable aspects	Privacy and confidentiality Less discrimination and stigma Increased access to comprehensive PHC package No intra-facility cross-referrals
	Unfavourable aspects	Waiting times for patients Poor patient care Patient dissatisfaction

ART, antiretroviral treatment; PHC, primary health care.

**TABLE 2:** Patient experiences of antiretroviral treatment services and care.

Themes	Sub-themes	Categories
Health system-related quality-of-care issues	Issuance of ARVs	One month's versus multiple months’ supply Collection of medication: self or third party Availability of medication
	Organisation of clinic schedules and care processes	Routine monitoring tests Appointments Filing system Waiting times
Patient experiences of ART services	Satisfaction with services	Confidentiality Discrimination
	Patient-centred care (staff attitudes and behaviour)	Respect for patients and privacy Punctuality Communication with patients
	Suggestions for process improvement	Issuance of ARVs: Fast queue for medicines Three months’ medicine supply Third-party medicine collection
		Organisation of clinic schedules: Early opening of clinic Meeting and collaborating with community Unannounced supervisory visits

ART, antiretroviral treatment; ARVs, antiretrovirals.

### Clinician perceptions of antiretroviral treatment integration into the primary health care package

Clinician perceptions of ART integration in PHC relate to the understanding of ART integration and factors that affect quality of care. The majority of participants shared some common understanding that ART integration meant a ‘supermarket approach’ (F1), patient-centred care, multi-purpose, accessible comprehensive one-stop services for clients (WHO 2008). ART patients are treated in the ‘same queue, same consulting room, by same staff member’ (F1) and are being attended at ‘first point of contact at the primary health clinic’ (F3).

Two main themes emerged as quality-of-care factors: provider-related factors and work environment, and integration factors affecting client services. Table 1 gives a summary of the sub-themes and categories identified under each theme.

#### Provider-related factors and work environment

Provider-related issues commonly highlighted by participants were the workload, staff development and support for integration.

**Workload:** Participants’ high workload was reported to have a negative effect on the quality of care for ART clients: ‘Too much workload leading to unhappy patient[*s*] because now they have to wait longer’ (F4). Reasons for high workload included staff shortages and increased activities such as: ‘… [*C*]ounselling for … new and follow-up … clients’; ‘examination … routine investigations’; ‘amount of forms to be completed’; ‘management of side effect and complication’; and ‘self-dispensing and issuing treatment … from consulting rooms’ (F1).

With regard to staff shortages, clinicians commented on the difficulties in providing quality services such as ‘full examination’, ‘exclude opportunistic infections’ and getting ‘proper history information’ (F1). ‘This result[*s*] in poor patient care as the staff tries to push the queues’ (F3).

Participants from the facility with partially integrated services referred to the high workload and unmanageable responsibilities for only one nurse allocated to ART: ’… [*T*]he amount of work is extremely exhausting’ (F3).

Clinicians also perceived the increased numbers of patients to be as a result of ‘across-border’ clients, referrals from hospitals and the closing down of NGO facilities.

**Staff development:** The main challenges of integrating ART services for clinicians were ‘… most nurses are not well-trained’ (F1) and ‘… others not liking some services’ (F4). In the facility with separate services, clinicians focusing on ART services lacked PHC skills. In the other three facilities very few participants had had Nurse Initiated and Management of Antiretroviral Therapy (NIMART) training. Many participants also did not appear to be well informed regarding the monitoring tests according to the national ART guidelines. This is how one participant commented on the situation: ‘If all the staff in the clinic can be properly trained on ART … patients will benefit … waiting times will be shorter than having to wait for only ART sister’ (F1).

**Support for integration:** Most clinicians reported good relationships between colleagues, except those in the facility with separate services, where there was a general feeling that management was not supportive enough of ART integration: ‘I think HIV programme coordinators and managers could do regular visits to assess the working conditions so as to know where they can assist’ (F4).

Lack of space in the waiting areas, especially during peak hours, was identified by the majority of clinicians in all facilities. Some participants considered the consulting rooms as ‘… adequate and fairly equipped’ (F1) whereas others considered them as poorly equipped, with ’… no bulbs for light’, ‘… poor ventilation, uv [*ultraviolet*] lights not working, no windows, infection control poor’ (F3), and inadequate storage space for medicines in the ART consulting room.

One participant in the facility with partially integrated services felt that with the integration of ART services the space could be shared across programmes: ‘I think integration will be of importance because rooms are few’ (F3). Space challenges were blamed for the lack of a multidisciplinary team approach in the management of HIV in the same facility: ‘There are no multidisciplinary team members’ (F3).

Clinicians regarded the lack of support staff such as well-trained HIV counsellors, data capturers and pharmacy assistants as a demonstration of the lack of management support for integration of ART services: ‘We had a problem with admin clerk nobody wanted to give out the files’ (F4).

Clinicians in the two facilities under the auspices of Tshwane expressed great dissatisfaction about having to dispense their own prescriptions in consulting rooms and considered the process of issuing medicines as opportunity time that could have been used to improve the quality of care given to clients, and ‘… dispensing ART's in the consulting rooms rather than in the dispensary further stigmatises HIV’ (F1).

Dedicated ART clinicians from the facility with separate services reported poor support from their colleagues in the PHC services, such as negative, uncooperative attitudes and poor teamwork as the main reasons for failure to implement the planned full integration: ‘If it was initially started to be integrated with PHC, they would have long being used to ART clients’ (F4).

Lack of collegial support for the ART programme resulted in unnecessary referrals of patients that could have been managed by any skilled PHC clinician. Participants also reported unwillingness of health care workers to learn from their peers: ‘Integration is just said not practiced because there are still health workers not willing to learn from other knowledgeable ones’ (F4).

Clinician participants from the two Tshwane facilities described the weekly visits by the NGO doctors as inconvenient, as it did not contribute to the continuity of care: ‘An ARV [*antiretroviral*] trained doctor is available only on Thursdays, sometimes there is difficult cases not up for telephonic discussion. Now patient must be referred’ (F1).

#### Integration factors affecting client services

The factors affecting client services can be divided into favourable and unfavourable aspects of integration (see Table 1).

**Favourable aspects of integrated services:** Clinicians from the three facilities with fully or partially integrated services highlighted the promotion of privacy and confidentiality as a result of integration: ‘Integration of services decreased the discrimination and stigmatisation because nobody knows the reason of visit’ (F1).

The majority of clinicians from the facility with partially integrated services reported that this model of care promoted discrimination and stigmatisation: ‘Those on ART are taken to a specific room other patients knows that room is for ART’ (F3). One clinician indicated that, ‘Comprehensive patient care improves team effort, nurse patient relationship, reduces frequent visit’ (F3).

Privacy was also perceived to be compromised in the separate ART facility where clients sometimes waited outside because of limited space in the waiting area. There was reference to clients defaulting on their treatment because of long queues, discrimination and stigma attached to the service: ‘Clients interrupt treatment because of long queues. Others cannot comply with treatment because of discrimination’ (F4).

According to the clinicians, the integration of services improved access to the comprehensive PHC package: ‘Integration helps e.g if they come for chronic/curative they also access ART service during that visit’ (F3). Comprehensive care in the fully integrated facilities also eliminated cross referrals between ART and PHC services on the same premises. Cross referrals were reported to sometimes contribute to ineffective patient care: ‘Not all personnel refer patients to respective area, documentation of referrals not emphasized’ (F4).

**Unfavourable aspects of integrated services:** Participants reported client ‘… waiting time is the problem’ (F3) for rendering quality services. One of the reasons for the increased waiting time was the longer duration of consultations: ‘There are much more to do with ART patient than … in a new diabetic …’ (F1) and ‘… [*C*]ontinuous counseling these patient needs and their long term emotional status’ (F3).

Introduction of additional services into PHC services that have ‘… not been accompanied by increase in personnel’ (F1) was reported to affect patient care negatively. Some clinicians reported difficulty in managing patients because of the alleged high patient load and lack of skills and competencies: ‘The quality of services may be compromised, as staff is rushing … through all the patients, some staff members … not trained in ART … might miss certain issues/conditions’ (F1).

A few clinicians from the facilities not fully integrated believed that ART integration into the PHC package caused patient dissatisfaction because it threatened their privacy and convenience: ‘Some patients are not happy and they want to be treated separately’ if they cannot get ‘… help right away’ (F4).

### Patient experiences of service and care

The two main themes that emerged from the patient FG interviews were health system-related quality-of-care issues and patient experience of ART services. Table 2 gives a detailed summary of identified themes, sub-themes and categories.

#### Health system-related quality-of-care issues

Issuance of ARVs and the organisation of clinic schedules were the two sub-themes identified in relation to the health system's effect on patients and their care. Most of the comments related to dissatisfaction and the potential impact on adherence. Patients also expressed their preferences and offered recommendations for improvement of services.

**Issuance of ARVS:** Patient participants in the fully and partially integrated facilities preferred receiving a multiple-month supply of medicine, as was done at other facilities: ‘But [*the*] problem is work. Can you give us treatment for three months?’ (MF1). Reference was made to the requirement of monthly collections of medicine having caused some clients to have lost their jobs: ‘They expelled me from work for coming to the clinic to collect my pills’ (FF4). In two facilities, participants expressed dissatisfaction that clients’ families were not allowed to collect their monthly supply of medication if they were unable to attend: ‘I was working and sent my child to collect some pills for me but they turned the child away’ (FF4).

Participants reported that although they received medicines on a monthly basis, some had from time to time received a short supply because of insufficient stock.

**Organisation of clinic schedules and care processes:** Participants in all facilities reported that monitoring tests were performed regularly and that test results were communicated to them.

The appointment and filing systems were sources of dissatisfaction for participants from the facility with separate ART services. Clients without appointments were attended to after the patients with appointments, which could result in waiting for almost the whole day: ‘I just came for the doctor to complete the social grant forms, now I have to wait until everybody [*has been served*]’ (MF4).

The different type folders for the clinical records of ART patients compared to other PHC patients in the same facility was considered stigmatising and discriminatory: ‘Their files differ with that of other patients. Some patients [*are*] diagnosed [*as*] ART patient by their files’ (FF4).

Participants in the three large facilities raised concerns about long waiting times, despite their having arrived early. Waiting the whole day to just collect medicine or being requested to return the next day for a due consultation was a frequent occurrence: ‘You arrive early at 8h45; maybe you will be called at 15h00 or … just about to go off’ (FF1) and ‘I arrived around past six in the morning … but we can go back home without being attended to’ (FF4).

In the fully and partially integrated facilities counselling on treatment compliance, possible side effects and safe lifestyles was ongoing. Clients in the facility with separate ART services indicated that clinicians focused on pushing queues and paper work and had no time for good patient care: ‘You report the rash to the sister; they don't even examine you but just write and thereafter you are given medicines without knowing the kind of rash being treated. They don't care’ (FF4).

#### Patient experiences of antiretroviral treatment services

Patients’ experiences of ART services in PHC clinics are divided in three sub-themes: satisfaction with services, patient-centred care (focussing on staff attitudes and behaviour), and suggestions for process improvement.

**Satisfaction with services:** Clients in three health facilities were satisfied with integration, as the organisation of services and confidentiality prevented stigma and discrimination and afforded ART patients the opportunity to mix with other patients in the facility: ‘I think the system is good because … you have to be treated with every one’ (MF1). Participants in the facility with separate ART services resisted integration of ART with PHC services and preferred to remain in the park home: ‘When we started they mixed us with other patients in the PHC clinic … We fought until we went back to our place’ (FF4).

**Patient-centred care:** The majority of participants in the fully and partially integrated facilities reported that clinicians ‘… [*T*]reat us with respect’ (FF3), although some participants from the separate services facility also felt that they were being treated with ‘… [*R*]espect, privacy and confidentiality’ (FF4). Others referred to rude staff and unfriendly attitudes. Interruptions of consultations with clinicians being called away to attend to other matters were seen as an infringement of privacy and also increased the waiting time for clients: ‘While you are busy being assisted, the sister is requested to come and help or attend to something’ (FF4).

Client participants at the same facility also referred to the absence of punctuality: ‘They have to keep [*on*] time to start working’ (FF4). They were also dissatisfied with poor staff communication regarding delays in patient consultations: ‘If they communicate to us that they are now going on lunch or tea break we can also be able to go to the gate and buy ourselves something to eat’ (FF4).

**Suggestions for process improvement:** Regarding the issuance of ARVs patients suggested a ‘… fast queue for medicines’ (FF2), ‘… two to three months’ treatment’ (MF1), and ‘… allow others to collect medicine for others’ (FF4). Patient recommendations for the organisation of clinics included early opening of the clinic ‘… so that sometimes when it is raining you can wait inside’ (FF1), ‘… meetings with the community‘(FF2), and unannounced supervisory visits.

## Ethical considerations

The study was approved by the Research Ethics Committee of the Faculty of Health Sciences of the University of Pretoria and the Tshwane Regional Research Ethics Committee. Permission to conduct the study was also granted by the operational managers of the four clinics. Participation was voluntary and all participants signed informed consent. Clinician questionnaires were completed anonymously and were only identified by the facility name. To ensure confidentiality, all hard copies of documents were kept in a locked cupboard, whereas a password was included in the electronic transcript files.

Justice was promoted by including providers and users of PHC and ART services in the study. Provider anonymity protected clinicians who may have expressed negative sentiments and also prevented any potential penalisation. Patients were not in any way influenced by the researchers to participate. Recruitment and data collection was accompanied by a respectful attitude towards the vulnerability of participants. Patients were also assured that non-participation would not influence their treatment in any way.

## Trustworthiness

The trustworthiness criteria of credibility, transferability, dependability and conformability (Lincoln & Guba 1985) applied to this study. Credibility was ensured through purposive sampling and the selection of health facilities that met the inclusion criteria. Two different methods of data collection and two different participant groups enabled triangulation. Transferability was promoted by the distinction between different models of integration of ART services and by verbatim quotations linked to facilities following different forms of integration or non-integration. This could assist the reader to understand the context and to decide on the applicability of the findings to other similar settings. To ensure dependability of the findings, a detailed description of the research design, methods, data analysis and ethical processes has been given. To enhance conformability, themes deducted from the data were checked with some clinician participants to verify data interpretations.

## Discussion

The findings of the study confirm many issues raised in previous studies. The inclusion of settings following different models of integration in this study provides more nuanced insights into specific factors related to fully, partially and non-integrated ART services and to services managed by different health authorities (local and provincial). The findings could also inform endeavours to strengthen the operational management of health facilities.

This study employed two data collection methods with two different groups of informants. Some perceptions and experiences of the integration of PHC and ART services were highlighted by both clinicians and patients. Two issues influencing satisfaction with client services that were considered important by both informant groups were patient privacy, confidentiality and discrimination, and waiting times. Other important findings relate to work environment, staff development, patient access to comprehensive (integrated) PHC, and experience of patient care.

### Patient privacy, confidentiality and discrimination

Participants in this study highlighted the reduced discrimination and stigmatisation of PLWHA that is associated with fully integrated services. Serenata (2010:28) describes integration as a way of promoting quality, non-discriminatory care and reducing the stigma attached to HIV and/or AIDS care services, whilst Uebel et al. (2013a:8) found that, despite patients’ preference for a clinician specialising in HIV care, they also had concerns about stigma associated with accessing ART from separate services.

### Waiting times

Similar to findings from other studies (Uebel et al. 2013b), ART integration and quality of care rendered in the PHC clinics was negatively affected by the high influx of patients to some of the Tshwane health facilities from across catchment borders, and staff feelings of being overwhelmed. Overcrowded facilities are a common phenomenon that is uncomfortable to both clients and clinicians (Xaba, Peu & Phiri 2012:540*),* whereas other clinics remain underutilised.

The level of patient satisfaction has been linked to patients’ retention in ART care (Wanderler et al. 2012:14). Despite prolonged waiting times, patients in our study were satisfied with the quality of care received (see also Chimbindi, Bärnighausen & Newell 2014:8; Kinkel et al. 2012:228; Wouters et al. 2008). Reducing waiting times for clients through queue management systems and the monitoring of waiting times is one of the national standards for quality patient care (NDOH 2011:7).

### Work environment

In this study clinicians perceived the following factors as part of an unsupportive work environment: lack of management support; inadequate infrastructure; shortage of support staff; high workload; overworked clinicians; lack of certain skills; negative staff attitudes; and long patient waiting times. These factors were all linked to additional responsibilities associated with ART integration into PHC services, a trend supported by findings from other studies (Davies et al. 2013; Hall 2004; Uebel et al. 2013a). Some studies have found that an unsupportive work environment increases stress (Hall 2004:32). Persistent high staff workload and lack of equipment predisposes clinicians to exhaustion, burnout, stress-associated illnesses, and absenteeism (Davies et al. 2013:2; Hall 2004:33). These conditions can lead to employees resenting ART or even forcing them to terminate their services despite enjoying their work (Hall 2004:32). A high clinician workload can undermine patient care processes and quality care and presents a hindrance to integrated ART services (Davies et al. 2013:3; Uebel et al. 2013a:6).

According to Uebel et al. (2011:2), the roll-out of ART integration worsened the poor work environment situation, especially in partially integrated facilities with one dedicated nurse for ART services (Davies et al. 2013:4). Although ART integration is the ideal for comprehensive care of PLWHA, it resulted in unintentional consequences such as an overflow of patients in facilities, increased waiting time, the worsening of an already stressful working environment and reduced quality of patient care.

### Staff development

Only a few clinicians in this study had been trained in NIMART. Lack of skills has also been reported to be used as an excuse for poor teamwork and poor support by colleagues for ART designated clinicians in partially integrated services (Davies et al. 2013:3). Heunis and Schneider (2006:257) emphasise multi-skilled nursing staff in key PHC programmes as an important prerequisite for implementing the PHC package effectively. For integration to be successful, Uebel et al. (2013b:3) recommend the streamlining of training in PHC courses, such as PALSA PLUS, with NIMART.

Shortage of skills at PHC level is a long-standing challenge identified in the Gauteng Departmental Strategic Plan for 2003–2004 (Gauteng Department of Health [DOH] 2002:5). This shortage relates to the inability of facilities to release clinicians to attend courses, especially those of longer duration (see also Wouters et al. 2011:804). Other studies identified that lack of skills increased waiting times for clients. Less competent clinicians had to consult knowledgeable ones repeatedly – a time-consuming and interruptive process (Heunis & Schneider 2006:258).

### Patient access to comprehensive primary health care

Clinicians in our study highlighted improved access to ART services and the comprehensive care package resulting from the integration, an observation supported by the literature (Kinkel et al. 2012:228). Integration substantially increases access to care treatment and coverage of patients on ART (Xaba et al. 2012:543) and might reduce HIV-related mortality (Kerschberger et al. 2012:1). Uebel et al. (2013b:2) reported increased accessibility of ART services following integration, which could contribute to a reduction in maternal deaths (Gonzalez 2014) and TB mortality (Kerschberger et al. 2012:1) in South Africa. This is in line with observations of higher numbers of patients initiated on ART at semi-integrated facilities than at separate facilities (Louwagie *et al.*2010:1054).

In this study patient access in the two not fully integrated facilities could have been influenced by the cross-referrals of patients between PHC and ART services. Patient cross-referral can be a cumbersome and time consuming process, delaying ART initiation and losing patients before they access treatment and care (Mabitsi 2013:14).

Schwartz et al. (2012) reported a high rate of unplanned pregnancies amongst women of child-bearing age on ART with unmet contraceptive needs and suggested full integration of services to reduce maternal and child mortality and morbidity. Meyer et al. (2005:135) recommended that, where resource-limited facilities were unable to integrate all the services in HIV care and treatment, at least basic reproductive health services, such as appropriate contraceptive counselling and the management of unplanned pregnancies, should be included.

### Experience of patient care

Generally patients in the current study reported positive clinician attitudes, except patients in the facility with separate services. Poor relationships between patients and health care providers and patient dissatisfaction could also be barriers to future access to care and treatment adherence (Chimbindi et al. 2014:32; WHO 2003:12). Improving staff values and attitudes is one of the six national health priority areas identified by the South African Minister of Health to fast track improvement of quality of care (NDOH 2011:6). Clinicians from the facility with separate services highlighted lack of interest and reluctance from their colleagues to embrace the ART programme and the negative impact it had on patient care, a finding also supported by Uebel et al. (2013a).

Treating patients with respect, dignity and in a non-discriminatory manner is the natural duty and moral obligation of clinicians and should be part of their institutional duties to fulfil standard requirements (Landman 2011:21). Patients should be provided with information regarding their treatment and care in health facilities (NDOH 2011:18). Routine education and counselling to ART patients is a strategy to improve adherence (Mukone et al. 2011:19).

Negative staff attitudes affect communities’ access to health services and create a negative reputation for a health department. Dahab et al.'s (2011:53) study on reasons for discontinuation of ART cites poor patient-provider relationships and discrimination as the main reasons for patients discontinuing their treatment.

## Limitations of the study

The current study had a limited scope comprising only participants from four facilities. Findings can therefore not be generalised. Furthermore, the focus was limited to the two main role player groups at the point of care. As not all the processes involved in the integration of services were part of the focus of the study, some facets of service integration were not explored.

## Practical implications and recommendations

Some of the gaps identified in the study are currently being addressed by the NDOH (Du Toit 2014:38; Gonzalez 2013:11), whereas continued NIMART training has contributed to the further reduction of skill deficits. The following are a few practical recommendations:

- Revision of performance norms targets in terms of nurse-patient ratio to accommodate the labour intensive nature of ART consultations.
- Introduction of an in-service training model with an appropriate balance between staff training needs and service delivery needs.
- Provision of the necessary resources to enable facility managements to integrate health programmes in PHC facilities.
- Strengthening health facility operational management and supportive supervision for programmes.

## Conclusion

This study demonstrated some of the important issues for health care providers and users that relate to the way in which PHC and ART services are integrated. Although fully integrated services is the preferred model of care, aspects that need more attention in integrating ART into the comprehensive PHC package are management support from health authorities for health facilities, improved working conditions and appropriate staff development opportunities.
